# Pituitary dysfunction following mild traumatic brain injury in female athletes

**DOI:** 10.1530/EC-23-0363

**Published:** 2024-01-16

**Authors:** Lára Ósk Eggertsdóttir Claessen, Hafrún Kristjánsdóttir, María Kristín Jónsdóttir, Sigrún Helga Lund, Ingunn Unnsteinsdóttir Kristensen, Helga Ágústa Sigurjónsdóttir

**Affiliations:** 1 Faculty of Medicine, School of Health Sciences, University of Iceland, Reykjavik, Iceland; 2 Department of Emergency Medicine, Landspitali – The National University Hospital of Iceland, Reykjavik, Iceland; 3 Physical Activity, Physical Education, Sport, and Health (PAPESH) Research Centre, Sports Science Department, School of Social Sciences, Reykjavik University, Reykjavik, Iceland; 4 Mental Health Services, Landspitali – The National University Hospital of Iceland, Reykjavik, Iceland; 5 Department of Psychology, School of Social Sciences, Reykjavik University, Reykjavik, Iceland; 6 deCODE Genetics, Inc/Amgen Inc., Reykjavik, Iceland; 7 School of Engineering and Natural Sciences, University of Iceland, Reykjavik, Iceland; 8 Department of Medicine, Landspitali – The National University Hospital of Iceland, Reykjavik, Iceland

**Keywords:** hypopituitarism (HP), mild traumatic brain injury (mTBI), traumatic brain injury (TBI), sport-related concussion (SRC), female athletes, hyperprolactinemia

## Abstract

**Objective:**

Pituitary dysfunction following mild traumatic brain injury can have serious physical and psychological consequences, making correct diagnosis and treatment essential. To the best of our knowledge, this study is the first to study the prevalence of pituitary dysfunction following mild traumatic brain injury in an all-female population following detailed endocrinological work-up after screening for pituitary dysfunction in female athletes.

**Design:**

This is a retrospective cohort study.

**Methods:**

Hormone screening blood tests, including serum blood values for thyroid-stimulating hormone, free thyroxin, insulin-like growth factor 1, prolactin, cortisol, follicle-stimulating hormone, luteinizing hormone, estrogen and progesterone, were taken in 133 female athletes. Results were repeatedly outside the reference value in 88 women necessitating further endocrinological evaluation. Two of those were lost to follow-up, and further endocrinological evaluation was performed in 86 participants.

**Results:**

Six women (4.6%, *n* = 131) were diagnosed with hypopituitarism, four (3.1%) with central hypothyroidism and two with growth hormone deficiency (1.5%). Ten women (7.6%) had hyperprolactinemia, and four (3.1%) of them had prolactinoma. Medical treatment was initiated in 13 (9.9%) women. Significant prognostic factors were not found.

**Conclusions:**

As 12.2% of female athletes with a history of mild traumatic brain injury had pituitary dysfunction (hypopituitarism 4.6%, hyperprolactinemia 7.6%), we conclude that pituitary dysfunction is an important consideration in post-concussion care. Hyperprolactinemia in the absence of prolactinoma may represent pituitary or hypothalamic injury following mild traumatic brain injury.

**Significance statement:**

Mild traumatic brain injury (mTBI) has become a growing public health concern as 50 million people worldwide sustain a traumatic brain injury annually, with mTBI being the most common (70–90%). As studies on mTBI have focused on mostly male populations this study aims to explore pituitary dysfunction (PD) in female athletes following mTBI. To the best of our knowledge, it is the first all-female study on PD following mTBI.

The study found that 12.2% of the participating women had PD after mTBI. Six (4.6%) had hypopituitarism and ten (7.6%) had hyperprolactinemia. These findings suggest that PD following mTBI is an important consideration that endocrinologists and other medical staff working with athletes need to be aware of.

## Introduction

Traumatic brain injury (TBI) is divided into mild (mTBI), moderate (moTBI), and severe (sTBI) injury. The most common is mTBI (1) which is caused by mechanical force being transmitted to the brain by a blow to the head, neck or body, acceleration–deceleration movement, or forces from a blast injury. Symptoms can present immediately, within hours or days and may or may not include loss of consciousness for less than 30 min (2, 3). Furthermore, recent diagnostic criteria for mTBI include a Glasgow coma scale (GCS) score of 13–15 after 30 min from the head injury, post-traumatic amnesia for less than 24 h, and normal neuroimaging studies (3, 4, 5). Symptoms of mTBI often resolve within a few weeks although prolonged cognitive and psychological effects can occur (6, 7). Furthermore, it has been demonstrated that mTBI can lead to hypopituitarism (HP) with a prevalence of 13–48% (8, 9, 10, 11, 12, 13, 14, 15), making it an important consideration of post-concussion care.

It has been speculated that HP following TBI may be due to direct or indirect injury to the pituitary gland or hypothalamus with four possible mechanisms of indirect injury (16):

1. Vascular injury to arteries supplying the pituitary gland resulting in ischemic damage (17).
2. Neuroinflammation and cytokine release can occur following mTBI causing pituitary damage (17).
3. Autoimmunity may also have a role in indirect pituitary injury following mTBI, as studies have found anti-pituitary antibodies in patients with previous mTBI (18).
4. Uncontrolled release of excitatory neurotransmitters following injury may damage neuronal cells in the pituitary gland by affecting cellular permeability (19).

Undiagnosed HP following mTBI can have serious physical and psychological consequences depending on which axes are affected (20, 21). While untreated glucocorticoid deficiency (GCD) can be life-threatening, symptoms of growth hormone deficiency (GHD) may be subtle and overlaps with symptoms of mTBI causing diagnostic delay. However, if left untreated, GHD can lead to decreased quality of life (22), metabolic alterations (23, 24, 25), osteopenia and osteoporosis (23, 25), and increased risk of cardiovascular and cerebrovascular morbidity and mortality (24, 25, 26). As hormonal supplementation therapy (HST) can reverse the metabolic and psychological effects of GHD (23, 25) accurate diagnosis and treatment is vital. Furthermore, even subclinical untreated hypothyroidism can have serious effects such as increased cardiovascular morbidity, and mortality (27) and gonadotropic deficiency can impact fertility and cause comorbidities of decreased gonadal hormones. Earlier reports indicate that the somatotropic axis is most commonly affected by mTBI with a prevalence of 8–48% (8, 11, 12, 13, 14, 15, 28, 29). The somatotroph cells may be prone to vascular injury as they receive blood supply via the long hypophysial portal vessels that are especially vulnerable (30).

Contrary to other pituitary hormones, which can become deficient following mTBI, hyperprolactinemia (HPRL) can occur following TBI (mTBI, moTBI and sTBI) (15, 31, 32, 33, 34, 35, 36). Pituitary gland injury affects the dopaminergic inhibitory control of prolactin release, resulting in rising serum prolactin (s-prolactin) levels (30). Thus, HPRL may be a sign of pituitary or hypothalamic injury following TBI (36) and may be a marker of TBI severity, as it has been shown to correlate negatively with GCS (37).

Definite diagnosis of GCD or GHD involves stimulation testing, the synacthen test for GCD diagnosis (38) and the insulin tolerance test (ITT) for GHD and GCD diagnosis. Due to potential risks related to the ITT, a safer method such as the combined test with growth hormone releasing hormone and arginine (GHRH–arginine test) has been validated for GHD diagnosis (39, 40). As stimulation tests can be time consuming and expensive, a biochemical marker for GHD screening would be beneficial. Serum insulin-like growth factor 1 (s-IGF1) has been proposed as a screening tool for GHD as low s-IGF1 may indicate GHD. However, its use remains debatable as s-IGF1 within normal range does not exclude GHD (28, 40).

Female athletes remain an understudied population with regards to mTBI (41) and HP following mTBI even though they appear to be more susceptible to mTBI (42) and recovery time seems longer than with male athletes (43). The aim of this study was to explore pituitary dysfunction (PD) including HP in female athletes following mTBI in sport and, to the best of our knowledge, it is the first study to do so.

## Materials and methods

### Study design and subjects

This study is a part of more extensive research on female athletes. A comprehensive description of the population inclusion criteria has been published (44, 45, 46). The study included women aged 18–45 years currently active in or retired from soccer, handball, basketball, ice hockey and martial arts in Iceland. Of the 508 women included in part 1 of the study, 166 women accepted further participation in part 2 including a detailed psychological interview focusing on mTBI history and neuropsychological testing (44, 46). Following part 2, all 166 women were invited to participate in part 3 of the study, presented here, with 151 women (91.0%, *n* = 166) accepting, 15 women (9.0%) were lost to follow-up. In part 3, a physical examination was conducted by the same medical doctor (LÓEC) for all participants including a neurological examination, and hormonal screening blood tests (SBT) for possible PD. Hormonal evaluation of all pituitary axes was performed with SBT taken in 133 women (88.1%, *n* = 151), nine were lost to follow-up and nine were pregnant. All SBT results were reviewed by the same endocrinologist (HÁS). If SBT results were outside reference value (O-RV) in two or three repeated blood tests for each serum hormonal measurement, they were defined as abnormal (45).

**Figure 1:**
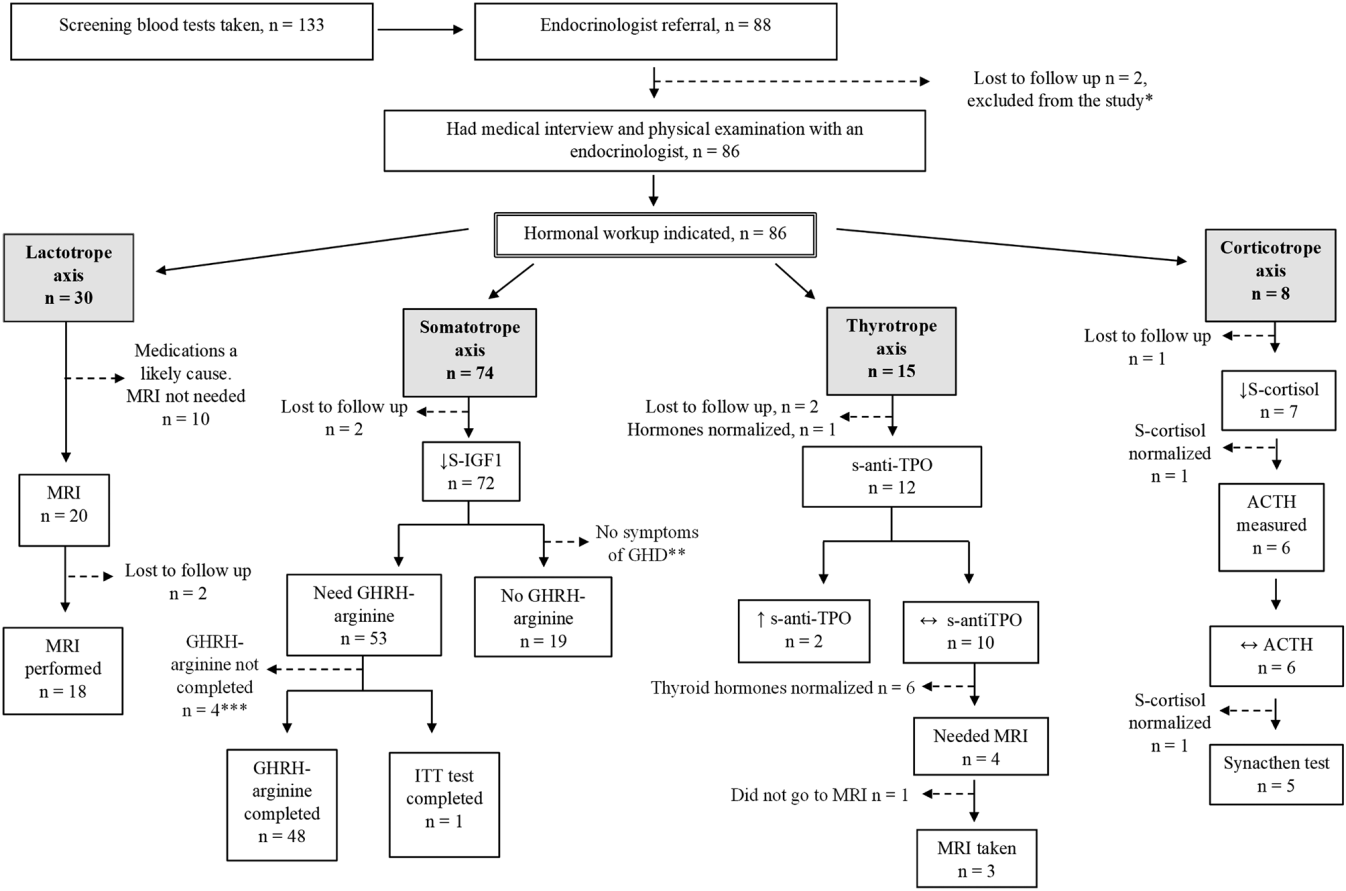
Overview of the study design and the detailed endocrinological work-up. *As two of the 88 women who needed further endocrinological evaluation did not attend the medical interview with the endocrinologist they were excluded from the study. Thus, 131 women instead of 133 had SBT and 86 women instead of 88 had a detailed endocrinological evaluation. **GHRH–arginine tests were not performed for 19 women who did not have symptoms indicating possible GHD. ***Lost to follow-up (*n* = 1), pregnant when the test was to be conducted (*n* = 2) and one woman is being treated for hyperprolactinemia and followed by the endocrinologist before the GHRH–arginine stimulation test can be performed if necessary. ↔ Blood test results within RV; ↑ Blood test results above RV; ↓ Blood test results below RV. ACTH, adrenocorticotropic hormone; anti-TPO, anti-thyroid peroxidase antibodies; GHRH–arginine, growth hormone-releasing hormone and arginine; ITT, insulin tolerance test; MRI, magnetic resonance imaging; S-cortisol, serum cortisol; S-IGF1, serum insulin like growth factor 1.

Of the 133 women who had SBT taken, 88 women (66.2%, *n* = 131) had results repeatedly O-RV necessitating further evaluation including a medical interview with an endocrinologist, physical examination and possibly further endocrinological testing (Fig. 1). Two of these 88 participants did not attend the visit despite repeated attempts to contact them and were thus lost to follow-up (Fig. 1). Thus, 86 women (64.6%, *n* = 131) attended the medical interview with an endocrinologist followed by detailed endocrinological testing as indicated. All female participants diagnosed with PD requiring treatment or follow-up will be followed by the endocrinologist (HÁS).

### Measurements and analytical methods

The SBT were taken at 08:00 h at the earliest convenient day for the participants and included serum thyroid-stimulating hormone (s-TSH), serum free thyroxin (s-fT4), s-IGF1, s-prolactin, serum cortisol (s-cortisol), serum follicle-stimulating hormone (s-FSH), serum luteinizing hormone (s-LH), serum estrogen (s-estrogen) and serum progesterone (s-progesterone). If the first SBT was O-RV for each hormone (Table 1), the blood tests were repeated for reevaluation.

**Table 1:** Hormone measurements and analytical methods of serum hormones measured.

Analyte	Assay name	Manufacturer	Instrument	CV%			Reportable range	Reference value	Specimen	
				Low contr	Med. contr	High contr			Type	Storage
TSH	TSH	Roche	Elecsys	11.1	1.3	2.2	0.005–100 mIU/L	0.270–4.20 mIU/L	Serum	+2–8°C
fT4	FT4 III	Roche	Elecsys	4.3	1.5	2.6	0.5–100 pmol/L	12–22 pmol/L	Serum	+2–8°C
Prolactin	Prolactin II	Roche	Elecsys	1.5	3.0	2.4	0.0470–470 μg/L	Female (not pregnant) 4.79–23.3 μg/L	Serum	+2–8°C
Cortisol	Cortisol II	Roche	Elecsys	5.4	1.5	1.6	1.5–1750 nmol/L	Morning (06:10 h): 133–537 nmol/L, afternoon (16:08 h): 68.2–327 nmol/L	Serum	+2–8°C
IGF1	IGF-I	Siemens	Immulite 2000	6.3	3.1	2.5	15–1000 μg/L	Manufacturer’s age-dependent reference range^a^	Serum	+2–8°C or −20°C
ACTH	ACTH	Roche	Elecsys	2.7	0.6	0.7	1.0–2000 ng/L	7.2–63.3 ng/L	Plasma	+2–8°C
GH	Growth hormone	Siemens	Immulite 2000	6.5	5.5	6.6	0.05–40 ng/mL	Females: up to 8 ng/mL	Serum	+2–8°C
Anti-TPO	EliA anti–TPO	Thermo Fisher	Phadia™ 250	–	–	–	–	<25 IU/mL = negative, 25–35 IU/mL = equivocal, >35 IU/mL = positive	Serum	+2–8°C

^a^Immulite product booklet, IGF-1, Immulite 2000 IGF-1 (PIL2KIGF-4, 2018–07-02).

ACTH, adrenocorticotropic hormone; Anti-TPO, anti-thyroid peroxidase antibodies; CV, coefficient of variation; fT4, free thyroxine; GH, growth hormone; High contr, high control; IGF1, insulin-like growth factor 1; Low contr, low control; Med. contr, median control; TSH, thyroid-stimulating hormone.

In the endocrinological interview, further information regarding mTBI history was gathered as well as information regarding previous medical history, medications and possible clinical symptoms of mTBI or HP. Thus, information on mTBI symptoms was gathered at four different times during the study period (part 1, part 2 and twice in part 3). Height, weight, blood pressure and heart rate were measured. Further endocrinological tests were then requested as necessary.

### Endocrinological tests

When s-fT4 was below reference value (RV) (Table 1) serum levels of anti-thyroid peroxidase antibodies (s-anti-TPO) were measured using the EliA method (fluoroenzymeimmunoassay) to exclude autoimmune hypothyroidism. Central hypothyroidism was suspected if s-anti-TPO was negative along with low or normal TSH levels and low s-fT4 levels. Consequently, magnetic resonance imaging (MRI) was requested for further work-up.

For s-IGF1, results below median RV (Table 1) were considered abnormal if clinical symptoms of GHD such as decreased vitality and energy, impaired psychological well-being (22, 25), and changes in memory and attention (47, 48) were also present and further evaluation with a GHRH–arginine test was performed as described in the consensus guidelines (40). If s-IGF1 was repeatedly below median RV without any symptoms of GHD, further endocrinological evaluation was not performed.

An ITT was performed in one woman due to practical reasons and strong clinical symptoms indicating GHD. It was performed as described in the consensus guidelines (40).

The lactotroph axis was evaluated using s-prolactin measurements*.* When s-prolactin level was found to be elevated, a macroprolactin analysis was performed to differentiate between monomeric prolactin and macroprolactin. When s-prolactin was repeatedly above RV (Table 1), an MRI was performed.

When s-cortisol was below 350 nmol/L (49), plasma adrenocorticotropic hormone (ACTH) was measured and a high dose (250 µg) synacthen test was performed as earlier described (50, 51). A normal response was defined as s-cortisol ≥440 nmol/L after either 30 or 60 min.

### Ethics

The study was approved by the National Bioethics Committee (no. VSN-18-091), the Icelandic Data Protection Authority, the Institutional Research Committee of Landspitali National University Hospital, Iceland, the chief medical officer of Landspitali National University Hospital, Iceland and Laeknasetrid outpatient clinic (OB/ei Tilv. 16).

### Statistical analysis

Statistical analysis was performed using R (version 3.6.1). A two-sample *t*-test and the chi-squared test were used to compare women with PD and women with normal pituitary function to identify possible risk factors for PD *(* Table 2). Categorical data were examined for association significance using Fisher’s exact test. The effect size (Table 2) was calculated using Cohen’s *d* for the two-sample *t*-test, Phi (ɸ) for chi-squared test with 2 × 2 contingency tables. Population size needed for 80% power was calculated using Lehr’s formula.

**Table 2:** Demographic and clinical characteristics of the study population. Women with pituitary dysfunction were compared to women with normal pituitary to identify possible risk factors for pituitary dysfunction. Statistical comparison between the two groups was not performed for sport, previous medical history, previous history of hormonal disease, hormonal contraception or menstrual changes as there were too few participants with PD for statistical analysis with the chi-square test. For the entire population (*n* = 131) the number of mTBI ranged from 1.0 to 4.0, BMI ranged from 19.1 to 46.5, time that passed from mTBI until SBT ranged from 0.04 to 35.2 years, the number of mTBI symptoms in endocrinologist interview ranged from 0 to 6.0, and the number of mTBI symptoms right after concussion from 1.0 to 8.0.

		Total *n* = 131	No PD *n* = 115	PD *n* = 16	Effect size	*P*
Sport^a^ (%)						
	Soccer	52 (40.3%)	45 (39.8%)	7 (43.8%)	–	–
	Basketball	12 (9.3%)	9 (8.0%)	3 (18.8%)		
	Handball	48 (37.2%)	44 (38.9%)	4 (25.0%)		
	Ice hockey	9 (7.0%)	7 (6.2%)	2 (12.5%)		
	Martial arts	8 (6.2%)	8 (7.1%)	0 (0.0%)		
Still playing (%)						
	Yes	71 (54.2%)	64 (55.7%)	7 (43.8%)	*0.08*	0.42
	No	60 (45.8%)	51 (44.3%)	9 (56.2%)		
Age (s.d.)		29.3 (7.6)	29.5 (7.7)	27.6 (7.5)	*0.25*	0.36
BMI (s.d.)		26.3 (4.7)	26.0 (4.5)	28.0 (6.2)	*0.42*	0.12
SBP (s.d.)		124 (12.1)	124 (12.4)	124 (10.1)	*0.00*	0.97
DBP (s.d.)		78.4 (9.1)	78.5 (9.1)	77.7 (9.4)	*0.09*	0.75
Previous medical history (%)						
	Yes	55 (42.0%)	51 (44.3%)	4 (25.0%)	–	–
	No	76 (58.0%)	64 (55.7%)	12 (75.0%)		
Previous hormonal disease (%)						
	Yes	13 (9.9%)	12 (10.4%)	1 (6.3%)	–	–
	No	118 (90.1%)	103 (89.6%)	15 (93.7%)		
HoC (%)						
	No	53 (40.5%)	46 (40.0%)	7 (43.4%)	–	–
	Before mTBI	21 (16.0%)	18 (15.7%)	3 (18.8%)		
	After mTBI	22 (16.8%)	21 (18.3%)	1 (6.3%)		
	Before and after mTBI	35 (26.7%)	30 (26.0%)	5 (3.1%)		
Menstrual changes (%)						
	Yes, after mTBI	16 (12.2%)	14 (12.2%)	2 (12.5%)	–	–
	No changes	115 (87.8%)	101 (87.8%)	14 (87.5%)		
Years from mTBI (s.d.)		5.1 (6.2)	5.2 (6.5)	4.3 (4.2)	*0.14*	0.58
Number of mTBI (s.d.)		2.2 (0.8)	2.2 (0.7)	2.0 (1.0)	*0.23*	0.40
Number of mTBI symptoms in endocrinologist interview (s.d.)		2.0 (1.5)	2.1 (1.5)	1.8 (1.4)	*0.20*	0.52
Number of mTBI symptoms right after concussion (s.d.)		3.4 (1.7)	3.4 (1.6)	3.5 (1.9)	*0.06*	0.86

^a^The total number of women who answered questions regarding the sport they participated in was *n* = 129, as two women did not report which sport they participated in. Of the 129 women, 113 had PD and 16 did not.

BMI, body mass index; DBP, diastolic blood pressure; HoC, hormonal contraception; mTBI, mild traumatic brain injury; PD, pituitary dysfunction; SBP, systolic blood pressure; s.d., standard deviation.

## Results

Of the 131 women who had SBT taken, 86 (65.6%, *n* = 131) had results repeatedly O-RV and were referred for further endocrinological evaluation. Following detailed endocrinological testing 16 women were diagnosed with PD (12.2%, *n* = 131), 115 had normal pituitary function. Population characteristics and comparison between the two groups are presented in Table 2 (see also Fig. 1).

Thyroid hormone levels were O-RV in 15 participants (11.5%, *n* = 131) and further work-up of the thyroid axis was performed in 12 (9.1%) of them (Fig. 1). Two of the 12 women had s-anti-TPO levels above RV and were diagnosed with autoimmune hypothyroidism. Six of the 10 women with s-anti-TPO within RV were not evaluated further as their thyroid hormone levels normalized during follow-up. Four women (3.1%, *n* = 131) were suspected having central hypothyroidism as they did not have s-anti-TPO and their s-TSH were low to normal with low fT4 levels. During follow-up of these four women, thyroid function tests were repeated at an interval ranging from 1 to 9 months with a mean of 3.2 months. Three of these four women had normal MRI results. One woman did not attend MRI and was lost to follow-up despite repeated attempts to contact her.

Of the 74 (56.5%, *n* = 131) women with s-IGF1 levels in SBT below median RV (Fig. 1), 19 did not have clinical symptoms indicating GHD and were not evaluated further. Clinical symptoms of GHD were present in 53 participants who were referred to further endocrinological testing (ITT *n* = 1, GHRH–arginine test *n* = 52). A GHRH–arginine test was completed in 48 of the 53 women (90.6%) (Fig. 1).

**Figure 2:**
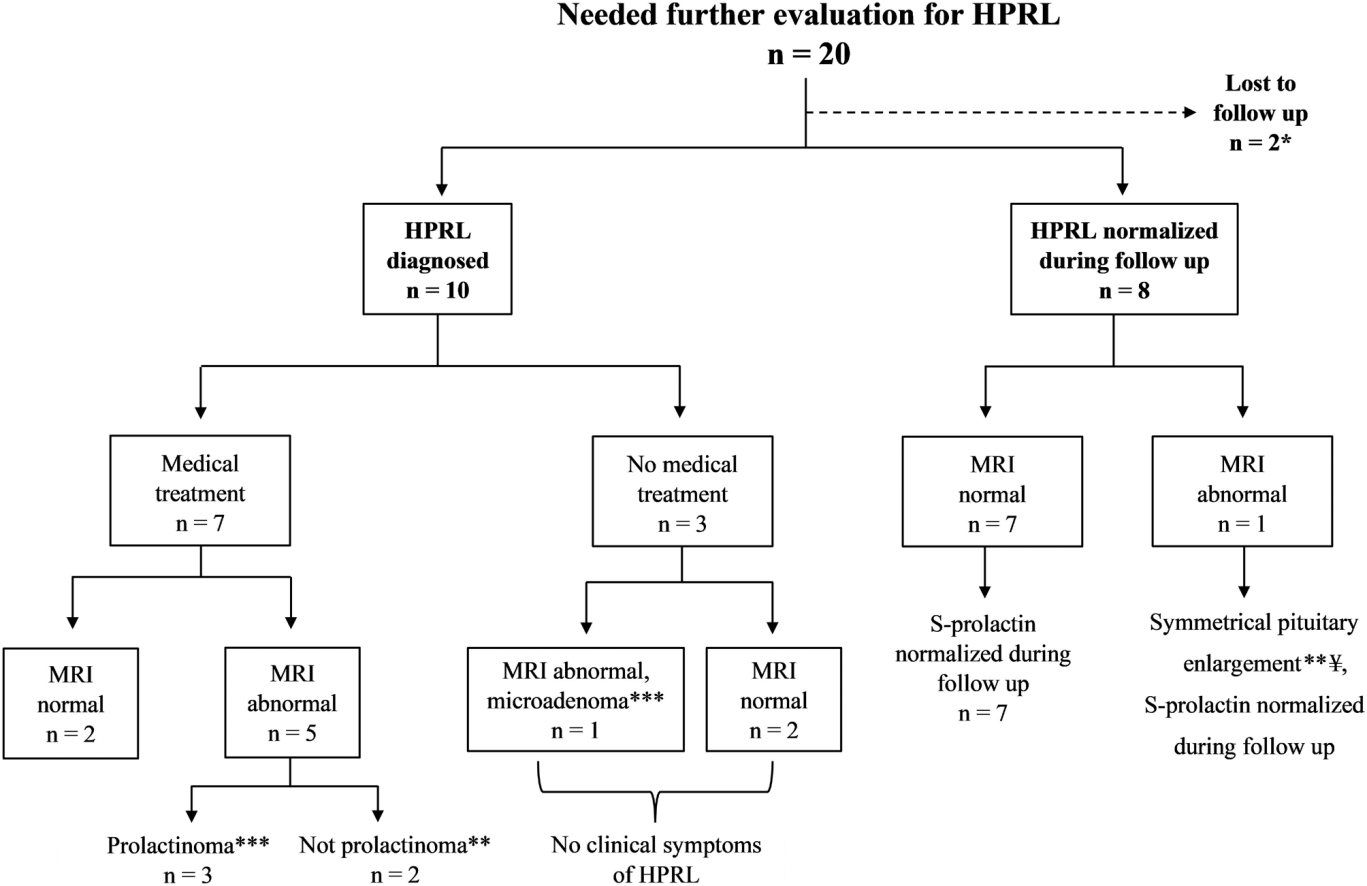
Results of the lactotroph axis evaluation. Ten women were diagnosed with HPRL, four of them were diagnosed with a prolactinoma. *Both women had elevated S-prolactin levels in SBT and normal MRI results. Both were lost to follow-up, one woman moved abroad and the other has not responded to repeated requests to repeat blood tests for follow-up. **Abnormal MRI without prolactinoma, total count *n* = 3: symmetrical pituitary enlargement *n* = 1 without a visible tumor, arachnoid cyst *n* =1, symmetrical enlargement of the adenohypophysis without a visible tumor (¥) *n* = 1. ***Prolactinoma, total count *n* = 4: microadenoma *n* = 2, regressing adenoma *n* = 1, cystic/hemorrhagic adenoma *n* =1. ¥ = symmetrical enlargement of the adenohypophysis without a visible tumor. HPRL, hyperprolactinemia.

Elevated s-prolactin was found in 30 women (22.9%, *n* = 131) (Fig. 1). Ten were taking medications that can cause HPRL (selective serotonin reuptake inhibitors (SSRI) *n* = 2, hormone contraception *n* = 5, SSRI and hormone contraception *n* = 3) (52). Thus, further work-up for HPRL was not indicated in those women. Of the remaining 20 women who had elevated levels of s-prolactin, 18 had an MRI of the pituitary gland and two were lost to follow-up (Fig. 1). Seven of the 18 women had visible changes of the pituitary gland (hypopituitary atrophy and signs of a regressing prolactinoma *n* = 1, microadenoma *n* = 2, cystic/hemorrhagic adenoma *n* = 1, concentric enlargement of the adenohypophysis with no visible tumor *n* = 1, concentric enlargement of the pituitary gland *n* = 1, arachnoid cyst *n* = 1) and 11 had normal MRI results (Fig. 2). Four (3.1%, *n* = 131) of the seven women with abnormal MRI results had a prolactinoma (hypopituitary atrophy and signs of a regressing prolactinoma *n* = 1, microadenoma *n* = 2, cystic/hemorrhagic adenoma *n* = 1) (Fig. 2). In summary, of the 18 women who had further work-up of the lactotroph axis, 10 women (7.6%, *n* = 131) had repeatedly elevated levels of s-prolactin and were diagnosed with HPRL. The s-prolactin levels normalized during follow-up in eight women who were not diagnosed with HPRL (Fig. 2). Four of the 10 women with HPRL were diagnosed with a prolactinoma and 6 women were not.

S-cortisol was below RV (below 350 mmol/L) in eight women (6.2%) (Fig. 1). Six were evaluated further with plasma ACTH measurements that were all within the RV. Five women needed further work-up with a synacthen test which was normal (peak s-cortisol ≥440 nmol/L) for all.

As has been reported, one woman had gonadotropin levels below RV in SBT. However, further endocrinological evaluation was not indicated as she was taking hormonal contraception (HOC) (45).

Following a detailed endocrinological work-up, 16 (12.2%) of the 131 participating women had PD. Six women (4.6%) had HP (GHD *n* = 2, 1.5% and central hypothyroidism *n* = 4, 3.1%) and 10 (7.5%) had HPRL (prolactinoma *n* = 4, 3.1% and HPRL without prolactinoma *n* = 6, 4.6%). No woman had more than one abnormal hormonal axes. Thus, PD was confirmed in 18.6% of the 86 women referred for further endocrinological evaluation. The mean time from the most recent mTBI until the endocrinological evaluation for the 16 women with PD was 4.3 years (Table 2) (min 2.4 months, max 15.3 years). The six women with HP had a mean time of 4.6 years (min 2.4 months, max 15.3 years) from the most recent mTBI until HP diagnosis was confirmed and the 10 women with HPRL had a mean time of 4.1 years (min 2.4 months, max 11.1 years) until HPRL diagnosis was confirmed. The time from mTBI until the endocrinological work-up for the two women who were diagnosed with GHD was 2 months for one of them and 15 years for the other.

No statistically significant difference was found between the 16 female athletes with PD compared with those with normal pituitary function concerning prognostic factors (Table 2). The effect size in the current study was largest for age, BMI, the number of mTBI, and the number of mTBI symptoms in the endocrinological interview (0.25, 0.42, 0.23 and 0.20, respectively) (Table 2). The calculated population size needed for 80% power was 261 participants for age, 89 participants for BMI, 296 for the number of mTBI, and 394 or the number of mTBI symptoms in the endocrinological interview. When comparing the number of mTBI symptoms (≤3 symptoms or >3 symptoms) between the women with HP with those without HP, no statistical significance was found (*P* = 0.19). No statistical significance was found between women with and without HPRL with regards to menstrual disturbances (*P* = 0.289).

Medical treatment or HST was required for 13 (9.9%, *n* = 131) of the 16 women with PD, thus 81% of women with PD needed medical treatment. All six women diagnosed with HP were started on treatment with HST (levothyroxine for central hypothyroidism *n* = 4, somatropin for GHD *n* = 2). Seven of the 10 women with HPRL required treatment with a dopamine agonist (cabergoline). Two of these seven women had normal MRI results and five had abnormal MRI results (prolactinoma *n* = 3, no prolactinoma *n* = 2) (Fig 2). Three women with HPRL were asymptomatic and did not need medical treatment (Fig 2). One of them had a pituitary microadenoma and is being followed clinically and the need for treatment reevaluated as necessary.

## Discussion

We found that 12.2% (*n* = 131) of female athletes with a history of mTBI had PD (HP 4.6%, HPRL 7.6%). As 50 million people worldwide sustain a TBI annually, with mTBI being the most common (70–90%) (1), this is a very important finding. Moreover, around 1.6 to 3.8 million sport-related mTBI occur annually in the United States (53) and the incidence is likely underestimated (54, 55). This highlights the importance of evaluating pituitary function following mTBI, especially as symptoms of HP may overlap with mTBI symptoms and HP can be treated. None of the women diagnosed with PD in the current study had ever been evaluated for HP or HPRL despite prominent clinical symptoms following the mTBI which had occurred up to 15.3 years (mean 4.3 years) before the study.

PD was confirmed in 18.6% of the 86 women who had SBT repeatedly O-RV. As 81.4% of the women who were referred for further endocrinological evaluation, did not have PD (45), the question remains which women should be screened and evaluated further for possible PD following mTBI and when should this screening occur? Some studies suggest that increased TBI severity increases the risk of HP (10, 15, 56, 57). However, HP prevalence following moTBI, a more severe brain injury than mTBI, has been reported to be lower than following mTBI (9). Thus, this remains debatable.

We found 4.6%, or six of 131 women, to have HP which is lower than previously reported (13–48%) (8, 9, 10, 11, 12, 13, 14). All six women with HP were treated with HST and experienced symptom relief with treatment during follow-up. This lower prevalence of HP found in our study might be explained by the long interval from the most recent mTBI until the SBT were taken (45). A mean time of 5.1 years passed from the most recent mTBI until the endocrinological evaluation was performed for the entire population (Table 2) and 4.6 years passed from mTBI until the endocrinological evaluation for the six women diagnosed with HP. As it has been suggested that HP may improve with time (33, 58, 59, 60, 61), the extended time interval from mTBI until HP diagnosis may explain the results of our study.

Central hypothyroidism (*n* = 4) was the most common form of HP, followed by GHD (*n* = 2), whereas in previous reports GHD has been most common (9, 10, 12, 13, 14, 15, 33, 36, 62, 63). This is interesting considering the hypothesis that secondary ischemic injury of the pituitary gland may be a possible cause of HP following TBI (16). The TSH- and ACTH-secreting cells reside in the anteromedial portion and the central wedge of the anterior pituitary gland, and the growth hormone-secreting cells reside in the lateral portion of the anterior pituitary gland. As the anteromedial and central wedge of the pituitary gland receives its vasculature from both the long and short hypophyseal portal vessels it should be better guarded from ischemic injury than the lateral portion of the anterior pituitary which receives its vasculature from the hypophyseal portal vessels alone (16, 64, 65, 66).

The prevalence of GHD following mTBI in our study (1.5%, *n* = 131) was lower than the previously reported prevalence of 8–48% (8, 11, 12, 13, 14, 15, 28, 29). Studies have suggested that GHD may improve with time which may explain the lower incidence of GHD in our study. One study found that 53.8% of patients with GHD had recovered after 3 years from TBI, although it is also discussed that GHD may arise as time passes (59). However, it is interesting that the time from the most recent mTBI until the endocrinological work-up for the two women with GHD was 2 months and 15 years. Thus, one had only recently suffered a mTBI while many years had passed for the other. Another possible explanation for the lower prevalence of GHD is that it may be caused by hypothalamic injury rather than by injury to the pituitary gland itself. As the GHRH–arginine tests was the diagnostic test for GHD rather than the ITT, results may be falsely normal as the GHRH–arginine test does not evaluate possible hypothalamic dysfunction (67, 68, 69).

Ten women (7.6%) had HPRL comparable to previously reported HPRL prevalence of 3.8–16% following mTBI, moTBI and sTBI (15, 31, 32, 33, 34). A mean time of 4.1 years passed from the most recent mTBI until HPRL was diagnosed. Thus, s-prolactin seems to remain elevated for an extended period in some cases following mTBI rather than improving with time as has been suggested for HP (33, 70).

The study did not find hypofunction in the gonadotropic or corticotropic axis (45).

No significant prognostic factors were found (Table 2). Although this is a large study of female athletes after mTBI, our power analysis (Table 2) show that a larger population is needed to identify possible prognostic factors to make PD screening following mTBI more targeted.

### Strengths and limitations

To the best of our knowledge, our study is the first to report the prevalence of HP and HPRL following mTBI in an all-female study. This is a strength of the study as female athletes are an underreported population (41). A detailed endocrinological evaluation was conducted by the same endocrinologist and relevant testing were performed when indicated to confirm PD which is also a strength of the study. Furthermore, follow-up with the same endocrinologist was offered to all women who were diagnosed with PD.

It is a limiting factor that the study is retrospective as mTBI tends to be underreported (55, 71, 72, 73). Results from part 1 and part 2 of the study also indicate underreporting as mTBI reporting increased from 40.2% to 64.8% after participants read a definition of mTBI (44). The time from mTBI until the endocrinological evaluation varied between participants which may be a limitation of the study. However, it may also be a strength, as it gives an insight into the long-term prevalence of PD following TBI. Although the study population is larger compared to previous studies on HP following mTBI (8, 9, 10, 11, 12, 13, 14), an even larger study population is needed to identify possible prognostic factors for PD following mTBI. Limitations of the diagnostic methods for possible central cortisol deficiency also need to be considered. Although measurements of s-cortisol and the high-dose (250 µg) synachten test were performed at 08:00 h, it is possible that variations in individual circadian rhythms could have resulted in false-negative results. Moreover, the use of the synacthen test rather than the gold-standard ITT for the assessment of the hypothalamopituitary–adrenal axis may not be reliable and can lead to false-negative results (74). However, as the ITT requires medical supervision, is physically demanding on patients, and can have contraindications, the synacthen test is often used as an alternative (75).

## Conclusions

We conclude that PD is an important diagnosis in post-concussion care as 12.2% of the female athletes (*n* = 16) had PD with 9.9% (*n* = 13) requiring medical treatment. Following mTBI 4.6% of the female athletes had HP which is lower than previously reported and may possibly be explained by recovery over time. HPRL may indicate pituitary or hypothalamic injury after mTBI as six of the 10 women with HPRL were not diagnosed with a prolactinoma.

## Declaration of interest

Sigrún Helga Lund is a statistician employed by deCODE genetics Inc./Amgen Inc.; she is not reimbursed for her work on this study. The authors have no competing interests to declare that are relevant to the content of this article.

## Funding

This study was funded by The Icelandic Centre for Research (grant number 207323-052) and Landspitali University Hospital Research Fund.

## Compliance with ethical standards

All procedures performed in studies involving human participants were in accordance with the ethical standards of the institutional and/or national research committee and with the 1964 Helsinki Declaration and its later amendments or comparable ethical standards. The study was approved by the National Bioethics Committee (no: VSN-18-091), the Icelandic Data Protection Authority, the Institutional Reasearch Commitee of Landspitali University Hospital of Iceland and Laeknasetrid outpatient clinic. Prior to participation, participants received information regarding the study design and gave their informed consent for participation and publication of the study results.

## Data availability

As the participants of this study did not give written consent for their research data to be shared, the data is not available.
